# Towards the Understanding of the Aging Behavior of p-PVC in Close Contact with Minced Meat in the Artwork *PO^E^METRIE* by Dieter Roth

**DOI:** 10.3390/polym15234558

**Published:** 2023-11-28

**Authors:** Paula Gassmann, Carolin Bohlmann, Valentina Pintus

**Affiliations:** 1 Institute for Conservation and Restoration, Academy of Fine Arts Vienna, Schillerplatz 3, 1010 Vienna, Austria; paula.gassmann@outlook.com (P.G.); c.bohlmann@akbild.ac.at (C.B.); 2 Institute for Natural Science and Technology in Arts, Academy of Fine Arts Vienna, Schillerplatz 3, 1010 Vienna, Austria

**Keywords:** PVC, plasticizer migration, DEHP, TCP, artificial aging, FTIR-ATR, Py-GC/MS, Dieter Roth

## Abstract

This paper presents scientific investigations into the materiality and aging behavior of a copy of Dieter Roth’s multiple *PO^E^METRIE* (1968), mainly made of p-PVC components and minced meat, with the aim of informing conservation-restoration strategies. The main issues were represented by plasticizer migration, fat diffusion, and leakage, which led to the formation of a sticky surface layer. Replicas of p-PVC without minced meat were prepared and artificially thermally aged, while several techniques were used to investigate both the artwork and the replicas in terms of materials and degradation state. These include UV/Vis imaging, pH measurements, FTIR-ATR, and Py-GC/MS. In addition to showing that p-PVC-based materials composed of slightly different plasticizers were affected by similar degradation pathways (i.e., plasticizer migration, yellowing, etc.), this study reports that fat components were also shown to be unstable, resulting in migration/leakage in different directions, where their degradation amplified that of the p-PVC bags. This work represents a first study of plasticizer migration and fat diffusion in the art and conservation context. Also, an ammine-wax type of lubricant was identified in the most recent p-PVC formulations as the replicas selected for this study, thus providing an important source of information in different polymer-based research areas.

## 1. Introduction

### 1.1. “PO^E^METRIE” by Dieter Roth—Challenges in the Preservation of Modern Polymeric Materials Combined with Natural Organic Fats

The preservation and conservation of contemporary art are especially challenging for conservators and conservation scientists because the artistic intention is far less clear than it might be for more traditional fields of art and needs to be questioned in far more detail. With some artworks, the idea as such is the core of the work; with others, it is the appearance or the materiality. Some artworks are supposed to look exactly the same as when they were produced, while others are allowed to undergo specific forms of decay. The list of ways contemporary artwork should be referred to is as long as the possible materiality or their combination can be made of. This can vary from inorganics to natural organics, synthetic organics, or even a mixture of all of them. Especially the combination of different kinds of material and how they were used (not always in the sense the material was supposed to be used from a manufacturing point of view) is making the preservation and conservation of contemporary art extremely complex.

Dieter Roth (1930–1998) in particular is no exception, but perhaps even one of the most extreme examples. The use of food as artistic material and sometimes the explicit intention of decay is bringing conservators partly to ethical conflicts but also to quite unique technical challenges.

**Figure 1:**
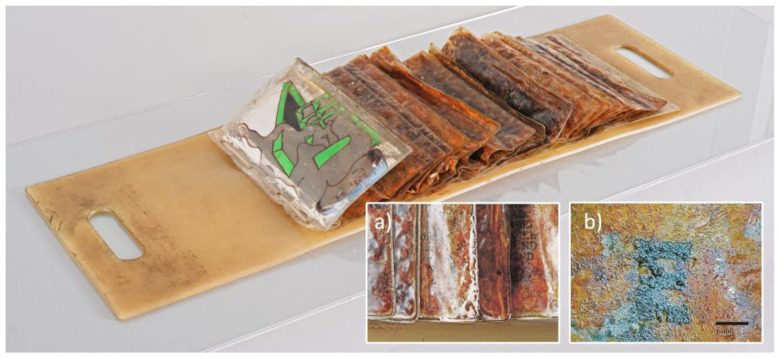
*PO^E^METRIE* (1968) by Dieter Roth, (**a**) sticky brownish surface layer on the bags and agglomeration of droplets towards the edges, and (**b**) text interspersed with exudates under magnification (raking light).

The Artwork *PO^E^METRIE* (1968) (Figure 1) is a special edition of Dieter Roth’s *POETRIE* Series, which he published between 1966 and 1969 as a biannual magazine. Next to the “normally printed” versions on paper, each volume was published additionally in the form of one or two special editions, which only vaguely reminded of a book in the conventional sense.

In the case of the following copy, Dieter Roth used a yellowish-translucent plate made of plasticized polyvinyl chloride (p-PVC) as the book’s cover, and transparent p-PVC bags are mounted on it as pages. The text of the 4th *POETRIE* volume is printed on the outside of the bags, while the bags themselves are stuffed with minced mutton (Figure 1). Over time, a sticky-brownish surface film formed on the bags’ outer surface (Figure 1a), in which not only dust and dirt particles could accumulate but also put the printed text at massive risk of being lost (Figure 1b). Although the discussion on artistic intention is cropped short here, the loss of the text would massively undermine the readability and the artwork as a book, and especially its characteristics as a special edition of the 4th edition of the *POETRIE* series. In several interviews and publications, Dieter Roth described himself primarily as a writer rather than an artist [1], giving his books and texts a special position in his oeuvre. The loss of the text therefore cannot be seen as intended by the artist, and conservation treatment appears reasonable. Since the combination of p-PVC and minced mutton is also rather unusual for contemporary art, several material-based questions need to be answered in more detail before a conservation concept can be established.

### 1.2. p-PVC Properties and Issues

Plasticized polyvinyl chloride (p-PVC)—widely present in modern and contemporary art collections as objects, clothing and footwear, furniture and upholstery, and housewares and toys—is known to deteriorate very rapidly in museum collections [2]. The tendency of colorless p-PVC-based objects to yellowing-browning in combination with stickiness and cracking are the main issues of this important plastic material, which require the attention of conservation scientists in cooperation with conservators, engineers, curators, and art historians.

PVC is obtained through the polymerization of the vinyl chloride monomer (C_2_H_3_Cl), which is usually produced from the reaction of ethene, chlorine, and oxygen. During polymerization, the polarity of the C-Cl bond increases the attraction of the individual polymer chains, resulting in a rigid and brittle material without additional components such as plasticizers. The properties of PVC can be greatly varied by the use of additives, making this material partially suitable for other uses. This diversity is also the reason for the widespread use of PVC. Additives for PVC include, above all, plasticizers, but also stabilizers, lubricants, pigments, fillers, blowing agents, fungicides, etc., which all have a significant influence on the chemical and physical properties of the resulting material. The exact recipes differ depending on the processing method and area of application of the later product [3,4,5]. 

In order to obtain a softer and more flexible product, the distance between the individual PVC polymer chains must be increased. This can be done by copolymerization with other polymers, e.g., polyvinyl acetate (PVAc) (called internal plasticizer), but also by simply mixing plasticizers with the PVC granulate (called external plasticizer). Over time, many different substances have been used as plasticizers, and for a long time, bis(2-ethylhexyl) phthalate (DEHP) has dominated the market. Next to DEHP, other plasticizers were used as well, including other alkyl phthalates, phosphates like tricresyl phosphate (TCP) or triphenyl phosphate (TPP), different polyesters, sebacates, and chlorinated hydrocarbons [3,5]. These external plasticizers are held in the polymer structure by secondary valency forces, which, however, also leads to a separation of the two substances over time and thus to the plasticizers being displaced from the polymer structure, which is widely known and described as plasticizer migration. The plasticizer migration takes place because, during aging, PVC and plasticizer become increasingly incompatible with each other, leading to the segregation and displacement of the plasticizer from the bulk material to the outer surface [3,4,5]. The loss of phthalate plasticizers as semi-volatile organic compounds (SVOCs) such as DEHP depends mainly on the temperature and air-flow conditions [6]. Room temperature and low air flow rates above the surface influence their evaporation from the surface, while higher temperatures and airflows promote diffusion from the bulk. DEHP has a boiling point of 386 °C and is prone to evaporate slowly from the surface under ambient conditions [2].

Other issues with PVC in terms of aging and degradation are related to the influence of light and heat, accelerated by the presence of oxygen. Compared with other synthetic polymers, PVC is less prone to photochemical degradation, so most of the decomposition phenomena are of thermal origin [3]. The heat-activated degradation starts at 83.6 kJ/mol, which is comparatively low energy (e.g., PE 192.5 kJ/mol, PS 230 kJ/mol, PP 272 kJ/mol) [3]. The thermal instability is due to structural irregularities in the material, particularly the -C=C- bonds at the chain ends. Tertiary carbon atoms, oxygen-containing compounds, and residues of catalyst from polymerization also play their part and reduce the resistance to thermal decomposition processes. Irregularities in the material with low binding energy (such as oxygen-containing groups, branching, or head-to-head connections) are easy targets where material degradation sets in first [3]. Cleavage of these low-energy bonds leads to radicalization of the polymer chain and the eventual elimination of HCl molecules. The splitting of a Cl atom not only leads to the formation of hydrochloric acid but also to a displacement of the electrons; as a result, conjugated double bonds are formed [3]. As soon as one HCl molecule is split off, further HCl molecules are separated like a zipper, meaning that this reaction continues autocatalytically [3]. From a number of 5 to 7 conjugated double bonds, the PVC shifts in color (from transparent to yellow, through red to brown, and finally turns black) [3,7]. The formation of these conjugated sequences can also lead to the formation of carbenium salts, which in turn lead to an intensification of the color depth (halochromic). This is particularly noticeable with PVC, which has already been thermally stressed [7]. However, in the context of the lifespan of p-PVC in indoor conditions, HCl elimination of the p-PVC takes place later than plasticizer migration, which will appear as the first issue in museum objects [6]. Investigations into the stability and degradation of PVC plasticized with DEHP highlight the impact of environmental conditions, and museum objects should be stored in closed environments, frozen, or kept at high relative humidity to prevent the loss of plasticizers [3]. Contact with materials like low-density polyethylene (LDPE) should be avoided due to the strong contact migration and diffusion of DEHP [3].

While all involved additives make the resulting plastic material a perfect fit for its intended industrial purpose, p-PVC as an artistic material has often been misused in its application. The same applies to the object on which this paper is based. Most plasticizers are well soluble in fats, and studies from the food packaging industry point out that plasticizers especially diffuse from the packaging into fatty foodstuffs [8,9,10,11]. Since diffusion is a bidirectional mechanism, this process also applies the other way around, and fatty components from foodstuffs will therefore also diffuse in and through the p-PVC. Next to plasticizer migration, fat diffusion needs to be seen as an essential part of the following case study and investigation. In addition to these two physical phenomena, the possibility of the bags leaking at the weld seams cannot be ruled out and appears to be a very likely additional factor that aggravates the problem.

According to the authors’ knowledge, so far, there have been no studies on plasticizer migration and fat diffusion in p-PVC associated with the fields of art and conservation. The only studies in this direction come from the food packaging industry, although the issue is viewed from exactly the other direction (the migration of packaging components into the food) [8,9,10,11]. Additionally, those studies refer to shorter periods of time, from a few days to a maximum of months, but nowhere near 60 years, as in the present artwork. Within the field of art and conservation, several different studies have been performed into the aging behavior of p-PVC [2,3,4,12], as well as on the impact of environmental [13] and storage conditions [14], or the cleaning of degraded p-PVC [14,15]. A recent review on PVC in plastic heritage collections reports the different degradation pathways and properties of PVC relevant to conservation, also highlighting several still existing gaps in understanding of PVC degradation, such as surface accumulation of plasticizers, degradation at temperatures below T_g_, etc. [6]. A number of different analytical techniques have been used to study plasticizer migration and degradation of p-PVC in art and conservation contexts [3,4,12,13,14,15,16] as well as for industrial purposes [17,18,19,20]. Degradation factors such as the yellowing of PVC in relation to dehydrochlorination and the length of the polyene sequence in PVC have been investigated by UV/Vis [12,21,22] and Raman spectroscopy [22,23,24], which have been considered suitable techniques for detecting polyenes and characterizing the current state of the material. The presence of phthalate in p-PVC has been investigated by Raman spectroscopy but also by Fourier Transform Infrared in Attenuated Total Reflection (FTIR-ATR), but the similarity of the di-alkyl phthalate spectra makes the exact identification challenging. On the other hand, FTIR-ATR represents, especially for art and conservation purposes, one of the widest-spread techniques. It is also one of the quickest but at the same time most informative ones for the identification of the major compounds and functional groups, as well as for the identification of specific surface phenomena [3,13,14,15,16]. It is generally complemented by Pyrolysis—Gas Chromatography/Mass Spectrometry (Py-GC/MS) for the precise identification and characterization of plasticizers and other additives [15,25,26].

### 1.3. Relevance to Other PO^E^METRIE Copies

Five further copies of the edition *PO^E^METRIE* could be visually examined during this research (Dieter Roth Foundation, Hamburg, Germany; LWL Münster, Münster, Germany; Staatsgalerie, Stuttgart, Germany; Kunstbibliothek, Berlin, Germany; Zucker Art Books, NY City, USA; and this copy, private ownership and permanent loan at Belvedere Wien, Vienna, Austria). The individual copies showed extensive similarities, but also individual differences. All copies have in common the format, the p-PVC components, and the text, but deviations can sometimes be found not only in the design of the book’s cover but also in the bag’s filling. Despite the minced meat commonly found in all copies, one copy seems to contain additional portions of fat (optically reminiscent of clarified butter) [27], while another copy appears to contain a higher content of blood in addition to the meaty component [28]. However, no analysis of the bag filling was performed, neither in this research nor for one of the other copies, because this would have required an opening of the bags for sampling. Therefore, it is unclear whether the bags are really stuffed with minced mutton or if Dieter Roth just claims that minced meat of another origin was used. In the present case, it does not appear as if blood was added, but differences in the fat concentration cannot be ruled out, also because natural products, especially those of animal origin, generally vary in exact composition. However, the differences mentioned did not necessarily lead to different material behavior, although the individual copies are in different states of preservation, and while some are still in good condition, others showed similar degradation patterns like those described here in more detail.

### 1.4. Aim of the Research

To establish preservation and conservation strategies for an artwork (e.g., suitable cleaning methods), deep knowledge of the materiality is generally required. Therefore, the following three steps were performed within this work:

1. Documentation and examination of the artwork—based on the localization and characterization of damage phenomena, material identification, and state of degradation.
2. Selection and preparation of replicas—based on the material identification of the artwork.
3. Artificial accelerated thermal aging studies—based on the prepared replicas with and without fillings to study material changes and interactions and on the difficulties in reproducing those specific phenomena.

For the artwork’s examination, a multi-analytical approach was chosen, including UV/Vis imaging, pH measurements, but also FTIR-ATR and Py-GC/MS analysis, while for the artificial thermal aging studies, replicas were examined mainly by optical observations and FTIR-ATR analysis.

## 2. Experimental Part

The three main experimental steps followed for this work, as described above in the aim of the research, are: (i) Documentation of the artwork—Methods; (ii) Selection and preparation of replicas; and (iii) Artificial thermal aging.

### 2.1. Documentation of the Artwork—Methods

#### 2.1.1. Photographic Documentation in Visible (Vis) and Ultraviolet (UV) Induced Fluorescence Light

Images in visible (Vis) and ultraviolet (UV)-induced fluorescence light were acquired to document the artwork’s condition and to obtain information about the materials and different surface phenomena. OMNILUX^®^ 36W G13 fluorescent tubes (Waldbüttelbrunn, Germany), which emit UV-A light in the 365 nm range, were used as the UV light source for the examination of the artwork. Photos were acquired using a digital SLR camera (Nikon Z7, Tokio, Japan); no additional filter was used.

#### 2.1.2. pH Measurements

The pH measurements were carried out using the agarose plug method, which was selected as a non-invasive method for the pH measurement of the material artwork’s surface. For this task, a thin foil of plugs was made using a concentration of 4% Agarose Low Melt ROTI^®^Garose (Carl Roth, Karlsruhe, Germany) in distilled water, as reported in the literature [29]. The then-prepared agarose plugs were applied to the artwork surface, left for 1 min in contact with it, and then placed in a portable LAQUAtwin B-172 pH meter (Horiba, Taunus, Germany). One drop of distilled water helped to increase the contact area. The pH meter is equipped with electrodes on one side and a digital LCD screen on the other side of the length. This pH meter allows the pH measurements of tiny samples or drops to be placed in contact with the electrodes in a relatively short time (approx. 15 s) and with high precision (pH ± 0.1).

#### 2.1.3. Fourier Transform Infrared Spectroscopy in Attenuated Total Reflection (FTIR-ATR)

Fourier Transform Infrared Spectroscopy in Attenuated Total Reflection (FTIR-ATR) analysis was performed for the material identification of the artwork. For this purpose, an Alpha-P FTIR Platinum ATR instrument (Bruker Optics, Ettlingen, Germany) equipped with a deuterated triglycine sulfate detector (DTGS) and a diamond crystal was used. Spectral measurements were taken between 4000 and 375 cm^−1^ at 4 cm^−1^ resolution. Measurements were done with 128 scans. While for optical examination and pH measurements, no sampling was necessary, for FTIR-ATR analysis, tiny samples were taken from the artwork (ca. 1 mm diameter), and the same samples could be reused for Py-GC/MS analysis. The resulting spectra were collected and evaluated with the spectrum software OPUS^®^ Version 8.0 of Bruker Optics, Germany, also including the Hummel Industrial Polymers 3.S01 Library and IRUG, 2009 (Infrared and Raman Users Group Spectral Database IRUG) library, and compared with literature. Additionally, pure PVC powder (CAS: 9002-86-2, Sigma Aldrich, Vienna, Austria), liquid DEHP (CAS: 177-81-7, Sigma Aldrich, Vienna Austria), liquid TCP (CAS: 1330-78-5, Sigma Aldrich, Vienna Austria), and ridged TPP pellets (CAS: 115-86-6, Sigma Aldrich, Vienna, Austria) were analyzed for comparison. The collected spectra were baseline corrected (concave rubber band method) and vector normalized.

#### 2.1.4. Pyrolysis—Gas Chromatography/Mass Spectrometry (Py-GC/MS)

Similar to the FTIR-ATR measurements, Pyrolysis—Gas Chromatography/Mass Spectrometry (Py-GC/MS) analyses were carried out for the material identification of the samples of the artwork (cover plate and bags) previously measured by FTIR-ATR. Measurements were done by using the Pyrolyzer PY-2020iD (Frontier Lab., Korijama, Japan) combined with a GCMS-QP2010 Plus (Shimadzu, Kyoto, Japan). The GC/MS unit was equipped with a capillary column SLB-5ms (Supelco, Bellefonte, USA) (30 m length × 0.25 mm ID × 0.25 µm film thickness) using bonded and highly cross-linked 5% diphenyl/95% dimethyl siloxane. The capillary column was connected to a Rxi Guard Column (Restek, Bellefonte, USA) deactivated silica guard column (5 m length × 0.32 mm ID). NIST 05 and NIST 05’s Library of Mass Spectra were available to identify the compounds, as well as the F-Search software (version 3.5.0, Frontier Laboratories Ltd., Korijama, Japan). For the Py-GC/MS analyses, sample material (around 0.22 mg) was put into a sample cup (ECO-CUP Frontier Lab, Korijama, Japan), and the pyrolysis temperature was programmed at 600 °C, while the pyrolysis interface and injector temperature were set at 280 °C and 250 °C, respectively. The temperature conditions of the GC column were set as follows: initial temperature of 40 °C, held for 5 min, followed by a temperature increase from 6 °C/min to 280 °C for 3 min. The helium gas flow was set to 1 ml/min, and the electronic pressure control was set to a constant flow of 31.7 mL/min in split mode at a ratio of 1:50. Mass spectra were recorded under positive-mode electron impact (EI) ionization at 70 eV and with MS interface and ion source temperatures of 280 °C and 200 °C, respectively. The mass spectrometer was scanned from *m*/*z* 50 to *m*/*z* 750.

### 2.2. Selection and Preparation of Replicas

#### 2.2.1. Complexity in the p-PVC-Based Replica Materials

Conservators and conservation scientists are well aware of the complexity of the problems involved in an individual artwork as well as the difficulties in applying any knowledge gained from studying and restoring one work to another artwork. The same applies to the transfer of test series based on replicas to a real work of art. Here, the difficulties already begin with the selection of adequate replica material. Replicas should be considered that have a chemical composition as close as possible to that of the considered artworks to better understand chemical reactions as examples involved in the degradation of the artwork and its physical consequences. Thus, the selection of suitable replica material is quite challenging since the exact formulations, as mentioned above, are subject to very rapid changes. The changes in the specific formulations are not only based on the fact that the different additives are intended to make the material suitable for a very specific purpose but are also associated with various governmental restrictions on chemicals due to recognized risks to human health and/or the environment. In the case of p-PVC, especially plasticizers like DEHP or TCP, which were once widely spread, are nowadays almost totally banned due to concerns for health and/or the environment [30,31] making it already quite challenging to find replica materials that have similar formulations to the artwork’s material. In addition to the selection of approximately suitable replica material, the reproduction of the specific damage phenomenon must be seen as the next challenge, which does not necessarily succeed. On top of this, the complex material combination of p-PVC together with organic fats (minced meat in this case) and their interaction with other factors like different soiling increase the difficulties even more in finding the right materials to study in experimental set-ups. Nevertheless, artificial accelerated aging studies on very similar materials in terms of chemical composition to those used for the artwork in general can already provide highly useful information about certain degradation mechanisms that can trigger damages to the artwork such as discoloration, cracks, loss of flexibility, or different separation processes (e.g., migration, diffusion), etc.

#### 2.2.2. Selection of p-PVC Replicas

Based on the similar chemical composition of the artwork’s materials—in accordance with the results obtained within this research—the following two types of materials were selected for this study and considered as replicas:

- Transparent Dia-slide cases (M67 ref.: 0111 _01_, PANODIA, Lyon, France) were purchased, containing bis(2-ethylhexyl) phthalate (DEHP) as a primary plasticizer and as repp material for the artwork’s bags. The Dia-slide sheets consist of two p-PVC foils, each with a layer thickness of 0.04 mm. The individual slots are separated by weld seams.
- No p-PVC containing TCP as plasticizer could be commercially found as replica material for the translucent yellowish covering plate. Because of the non-availability of tricresyl phosphate (TCP)-based p-PVC types of materials, a p-PVC containing triphenyl phosphate (TPP) (FB01//B2, JEDI Kunststoff GmbH, Eitorf, Germany) was instead selected. Possible differences between phthalate- and phosphate-based plasticizers were still studied. The JEDI plate has a thickness of 1 mm.

In contrast to the samples of the artwork—bags and cover plates—the replica materials will be referred to as Dia-bags and JEDI plates.

#### 2.2.3. Filling

For the Dia-bags, fillings were of interest for this research and were considered closely. To study the effects of the bag’s filling on the stability of the PVC bags, minced beef and clarified butter were selected as meaty and fatty components of animal origin. Although Dieter Roth claimed he used minced mutton for the filling, minced beef was chosen over minced mutton due to its easier availability. On the other hand, clarified butter was also considered because of its optical similarity to the fillings of the copy in Münster, Germany, but also because studies from the food packaging industry suggest an increased migration rate of plasticizers from the p-PVC when they come into contact with fatty foods of animal origin. Both were bought at a local Viennese grocery store in 2022.

#### 2.2.4. Methods

For the examination of the replica’s material, mostly FTIR-ATR (see Section 2.1.3) and Py-GC/MS (see Section 2.1.4) analysis were done, while for the artificial thermal aging studies, replicas were examined mainly by optical observations and FTIR-ATR analysis. The latter was used without the need for sampling and for monitoring and analyzing possible chemical changes on the p-PVC-based replica’s surface. Five measuring spots were chosen on each unaged and aged replica. The collected spectra were averaged (in the case of the replicas), baseline corrected (concave rubber band method), and vector normalized. On the other hand, further Py-GC/MS analyses on the aged replica material could not be performed because of technical reasons.

### 2.3. Artificially-Accelerated Thermal Aging

Artificial accelerated thermal aging was selected for the present case study since p-PVC is more prone to decomposition phenomena of thermal origin [3]. For this purpose, an aging oven (Universalschrank UN55plus, Memmert, Büchenbach, Germany) was used. While temperature and time can be set precisely, relative humidity (% RH) cannot be adjusted at this device. RH % in the oven was measured using a data logger (UX100 HOBO, onset, Bourne, MA, USA). During the aging tests, values between 30 and 10% RH were measured. Replicas, including the Dia-bags (without filling) and JEDI plates, were aged at 70 °C for up to 21 days and regularly controlled using optical observations and FTIR-ATR analysis. This temperature value was selected as a proper temperature for such studies [3].

## 3. Results and Discussion

### 3.1. Documentation and Examination of the Artwork

**Figure 2:**
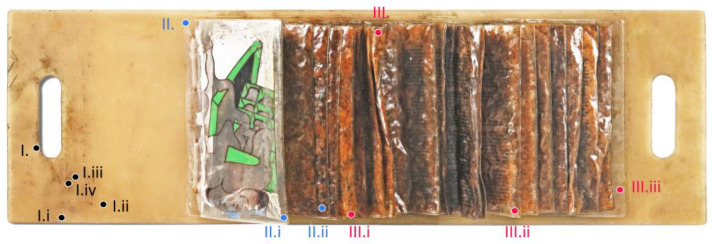
Overview image of *PO^E^METRIE* (1968) by Dieter Roth with the measuring spots, which are listed and described in Table 1. Spots labelled as I and black colored indicates the measuring spots selected on the plates (I., I.i, I.ii, I.iii, and I.iv); the ones as II and blue colored shows the spots on the non-sticky areas of the bags (II., II.i, II.ii), while those labelled as III and red colored are spots selected on exudate (III.) and sticky parts of the bags (III.i, III.ii, and III.iii).

As a first step in this research, the artwork was documented and closely examined using a multi-analytical approach. Visual examination and photographic documentation were performed in visible (Vis) as well as in ultraviolet (UV)-induced fluorescence light (UV/Vis-imaging) to distinguish and localize specific surface phenomena. pH measurements on different spots of the artwork’s surface were used to characterize those surface phenomena. Next to these non-invasive techniques, sampling was performed for FTIR-ATR and Py-GC/MS analysis, taken at representative areas. These minimally invasive techniques were used to identify and characterize the main materials used in the artwork and to investigate possible degradation pathways. Figure 2 gives an overview of the individual measuring points, while Table 1 lists the methods and results obtained for each of them. These findings are hereafter presented and discussed.

#### 3.1.1. UV/Vis-Imaging and pH Measurements

**Figure 3:**
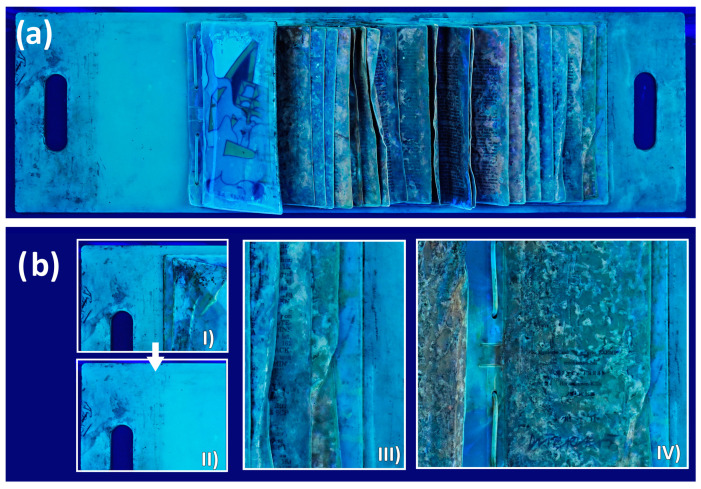
(**a**) General overview of the UV image of the artwork. (**b**) Images of some details of the artwork: (**I**) first bags open and continuing dark absorbing parts of “dry” soiling; (**II**) first bags closed, indicating a recognizable clear soiling edge and suggesting that the first bags were open over a longer period of time; (**III**) yellowish fluorescing droplets running towards the right carrying handle of the artwork, suggesting the artwork was presented or stored vertically hanged; (**IV**) last bag stuffed with minced mutton/imprint: agglomeration of yellowish fluorescing substance between the bags and droplets pointing towards leakage at the weld seam of the bags.

Images in visible (Vis) and ultraviolet (UV)-induced fluorescence light of the artwork highlighted different surface degradation phenomena and their distribution based on the different fluorescences. Within the UV images, especially, the difference between “dry/non-sticky” (more particle-based soilings) and “sticky” surface areas became obvious. The agglomerations of dust and particles exhibit a strong absorption of UV radiation and appear dark to black within the emission (Figure 3a,b). The sticky areas, on the other hand, showed a yellowish fluorescence, referring to organic fatty components (Figure 3(bIII,bIV)). Additionally, information about how the artwork was once presented or stored became apparent. On the yellowish covering plate of the artwork, a clear soiling edge on the left side is recognizable, which indicates that the artwork was exhibited or stored for a longer period with its first bags opened (Figure 3(bI,bII)). Under the last bag on the right side of the plate, yellowish fluorescent traces are running in the direction of the right carrying handle (Figure 3(bIII,bIV)). These indicate a vertically aligned presentation or hanging of the object, which was not clear and known from the historical documentation. The UV images also indicate that leakage from the bags, particularly at the inner weld seam, contributed to the formation of the sticky surface film (Figure 3(bIV)). However, this does not mean that fat diffusion can be ruled out. Due to the good clarity of the distribution of the different phenomena, the UV images were used to determine the significance of further measurement points.

The pH measurements side complement those optical observations by pointing out that especially parts with high levels of exudates and yellowish fluorescence (I.iv, III.i, III.ii, and III.iii in Figure 2) show especially lower pH values (between 3.9 and 4.6), referring to degradation and/or migration processes of acidic compounds. The lowest pH value of 3.9 was measured directly on a bigger droplet of exudates in the bags (spot III.i, Table 1 and Figure 2). Areas that did not exhibit a sticky surface layer showed higher pH values (I.i and I.ii 4.9 at the plate, II.i and II.ii 5.8–5.1 at the bags, Table 1 and Figure 2). The combination of UV-imaging and pH measurements gives a good overview of the distribution and characteristics of the different surface degradation phenomena. While the yellowish fluorescence in the UV images points toward organic fatty compounds, the pH measurements helped to characterize the exudates as highly acidic.

#### 3.1.2. Material Identification by FTIR-ATR and Py-GC/MS

##### Artwork’s Cover Plate and Cover Plate’s Exudate

**Figure 4:**
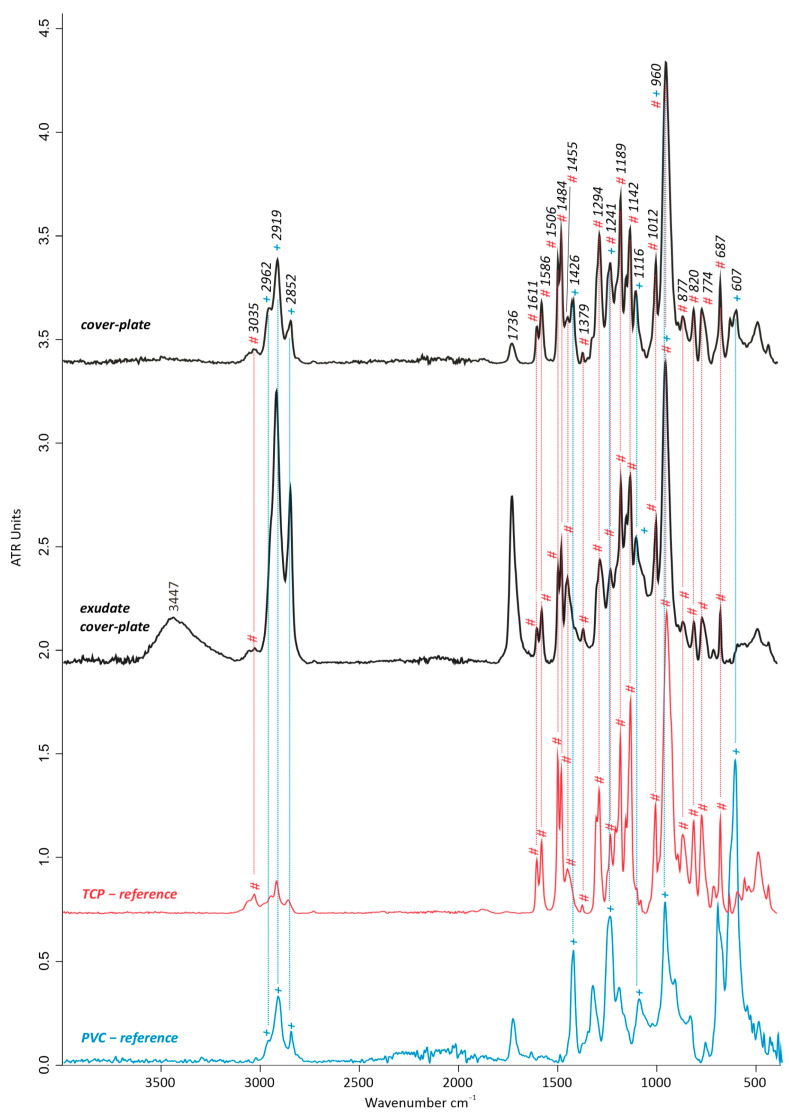
FTIR-ATR spectra of the artwork’s cover plate and the cove plate’s exudate, compared with the TCP (tricresyl phosphate) and PVC (polyvinyl chloride) reference spectra. + indicates the contribution bands of PVC, while # indicates TCP.

The material type of the artwork’s cover plate was identified through FTIR-ATR measurements as polyvinyl chloride (PVC) with tricresyl phosphate (TCP) as a plasticizer/flame retardant. The FTIR-ATR spectrum in Figure 4 of the sample analyzed (taken from spot I.—non-sticky, Figure 2) is characterized predominantly by the contribution of TCP. The peak at 3035 cm^−1^ shows the aromatic *v*(=C-H) complemented with those ring *δ*(C-H) at 877, 820, and 774 cm^−1^, while the peaks between 1611 and 1455 cm^−1^ and at 1379 cm^−1^ indicate aryl substituents and aryl CH_3_ vibrations [32], respectively. The *v*(P-O) of the phosphoryl groups can be seen at 1294 cm^−1^, while the peaks at 1189 and 1142 cm^−1^ are due to deformation vibrations of the aryls involved in plane C-H [32]. However, the peak at 1189 cm^−1^ can also refer to aromatic *v*(P-O-C) together with the one at 1241 cm^−1^ [32]. The latter is additionally showing the influence of the *δ*(CH-Cl) out-of-plane of PVC [3,19,33]. The peak at 960 cm^−1^ can be interpreted as an overlapping of the aryl vibrations of the PO [32] with the *δ*(C-H) out-of-plane in PVC [19,33]. Additional signals of the PO_3_ groups can be found at 1012 cm^−1^ and interpreted as their stretching vibrations [33].

Despite the strong dominance of the TCP plasticizer in the obtained spectrum, a few characteristic peaks for polyvinyl chloride (PVC) were also detected (Figure 4), in addition to those already mentioned at 1241 and 960 cm^−1^ overlapping with TCP. These peaks correspond to 1426 cm^−1^ *δ*(CH-Cl) and 607 cm^−1^ *v*(C-Cl) [19,33,34]. Furthermore, the peaks at 2964 *v*_a_(C-H) in CH_3_, 2924 *v*_a_(C-H), and 2867 cm^−1^ *v*_s_(C-H) in CH_2_ are ascribed mostly to PVC and to a lesser extent to TCP [17,18,19,32,33].

The FTIR-ATR spectrum of the sticky exudate sample (taken from sample spot I.iii, Figure 2) shows a broad agreement with the sample of the non-sticky part of the cover plate, thus indicating the main contribution of the TCP bands (Figure 4) and their exudation towards the surface. Nevertheless, the characteristic peaks for PVC, which were found in the non-sticky part of the cover plate, were here missing. Moreover, a higher relative intensity of the *v*(OH) band at ~3447 cm^−1^ and of the *v*(C=O) at 1736 cm^−1^ directs to higher alcoholic components and the formation of carboxylic acids. Both the increase of OH and C=O can be explained by the likely presence of grease accumulation in the part where the sample was taken and analyzed, which was clearly noticeable optically.

**Figure 5:**
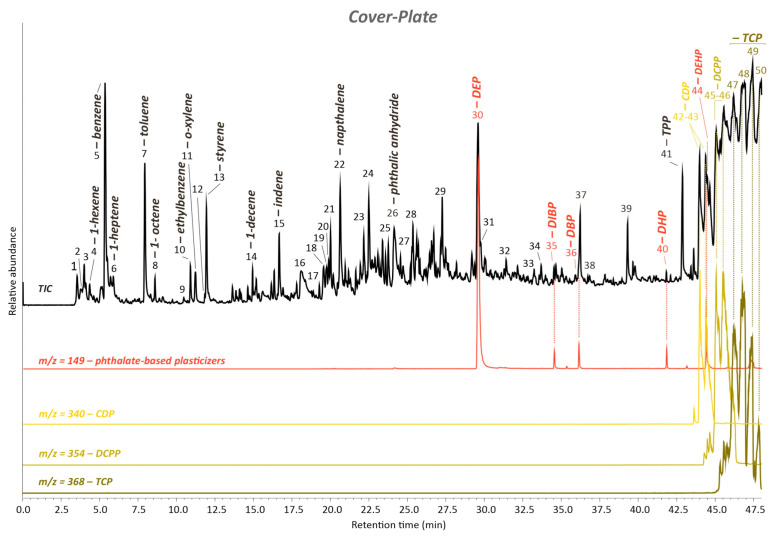
Total ion chromatogram (TIC) of the cover plate sample acquired by Py-GC/MS. The numbered peaks of the TICs are listed and described in Table S1. DEP: diethyl phthalate; DIBP: diisobutyl phthalate; DBP: dibutyl phthalate; DHP: dihexyl phthalate; DEHP: bis(2-ethylhexyl) phthalate; TPP: triphenyl phosphate. Extracted ion chromatograms (EICs) of the ion mass with *m*/*z* = 149 for phthalate-based plasticizers, *m*/*z* = 340 and 354 and 368 for cresyl diphenyl phosphate (CDP), dicresyl phenyl phosphate (DCPP), and isomers of tricresyl phosphate (TCP), respectively, are characteristic for the group of phenyl phosphate plasticizers/flame retardants.

The FTIR-ATR results for the non-sticky part of the cover plate were confirmed by Py-GC/MS measurements, and additional information could be obtained, mainly related to the presence of phthalates-based plasticizers and triphenyl phosphate (TPP) plasticizer/flame retardants. Next to the characteristic pyrolysis products of PVC (Figure 5, Table S1), including unsaturated and aromatic hydrocarbons like benzene (peak #5), toluene (peak #7), ethylbenzene (peak #10), o-xylene (peak #11), indene (peak #15), and naphthalene (peak #22) [35,36], several phthalates-based plasticizer pyrolysis compounds could be detected, otherwise unnoticeable by FTIR-ATR analyses.

The extracted ion chromatogram (EIC) of the ion mass with *m*/*z* = 149, typical for phthalate-based plasticizers, was obtained to highlight their presence in the analyzed sample and thus in the total ion chromatogram (TIC). This procedure showed the following products in Figure 5: phthalic anhydride (#26), diethyl phthalate (DEP) (#30) as the most abundant one, diisobutyl phthalate (DIBP) (#35), dibutyl phthalate (DBP) (#36), dihexyl phthalate (DHP) #40, and bis(2-ethylhexyl) phthalate (DEHP) (#44). In addition to those, two main phosphate-based plasticizers/flame retardants were identified and characterized, which are also shown in Figure 5 by their EICs: triphenyl phosphate (TPP) (#41) and tricresyl phosphate (TCP). The latter produced several and different pyrolysis products under the set-up conditions used, such as four main isomers (#47–50), as highlighted in the EIC at *m*/*z* = 368 (Figure 5, Table S1). Additionally, as shown in the EIC at *m*/*z* = 340 in Figure 5, two main peaks of cresyl diphenyl phosphate (CDP) (#42,43) type of additive were detected at RT 43.94 and 44.36 min. Following those, two peaks of a further additive such as dicresyl phenyl phosphate (DCPP) (#45,46) could be found at RT 45.07 and 45.54 min, as better evidenced in the EIC at *m*/*z* = 354 (Figure 5).

Finally, next to the phosphates and phthalates compounds, peak #13 gives evidence for the use of a third flame retardant such as benzenepropanoyl bromide, which overlapped with styrene (Figure 5, Table S1).

##### Artwork’s Bag and Bag’s Exudate

**Figure 6:**
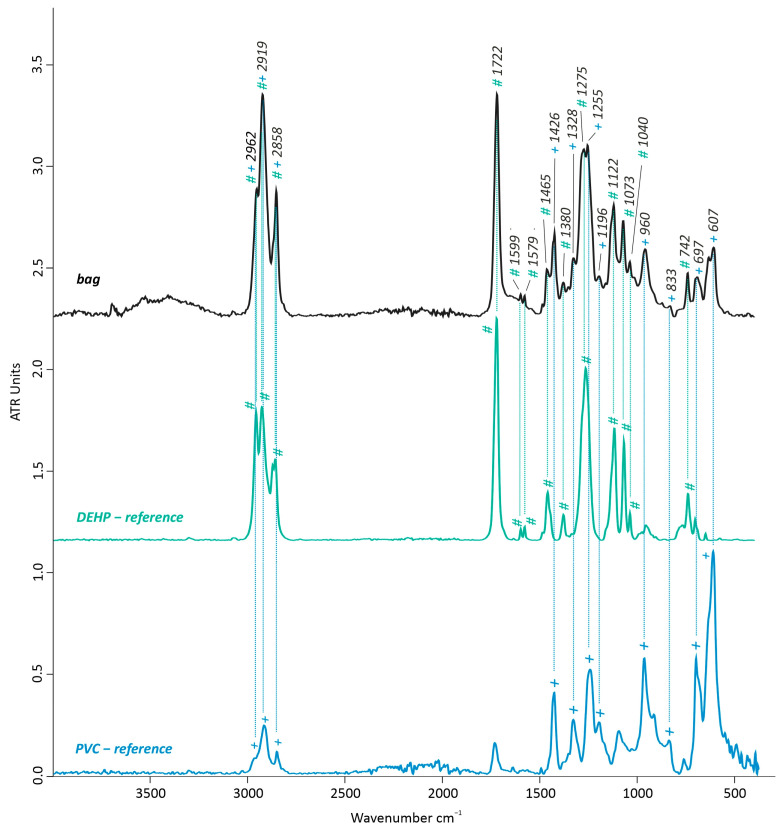
FTIR-ATR spectrum of the transparent bag’s sample of artwork compared with DEHP (bis(2-ethylhexyl) phthalate) and PVC (polyvinyl chloride) as reference spectra. + indicates the contribution bands of PVC, while # indicates DEHP.

The analyzed transparent bags of the artwork (taken from point II. non-sticky, Figure 2) were made of PVC plasticized with phthalate-based components according to the FTIR-ATR measurements. Particularly, a good match with the IR reference spectrum of bis(2-ethylhexyl) phthalate (DEHP) was registered (Figure 6). Nevertheless, it has to be considered that phthalate-based plasticizers generally present a highly similar IR spectrum, which does not allow us to distinguish them with absolute certainty. The intense peak at 1722 cm^−1^ of *v*(C=O) indicates aromatic esters of the plasticizers, while the peak at 742 cm^−1^ is related to *δ*(=C-H) out-of-plain of a 1, 2 disubstituted benzene ring. The double peak at 1599 and 1579 cm^−1^ refers to phthalate compounds due to *v*(C=C) [3,13,18,19,33,34] while the peaks at 1465 cm^−1^ *v*(C-O-C), 1380 *δ*(CH_3_), 1275 and 1122 cm^−1^ (CH aromatic in-plane deformation), 1073 cm^−1^ (CH or C-C aromatic in-plane deformation), and 1040 cm^−1^ *v*(C-O) can also be identified with phthalic acid esters [33].

In addition to the bands between 3000 and 2800 cm^−1^ *v*(CH), the typical peaks for PVC can be found at 1426 cm^−1^ *δ*(CH_2_-Cl), 1328 *δ*(CH_2_), 1255 cm^−1^ *δ(*CH-Cl) out-of-plain, and 1196 cm^−1^ *ω*(CH), together with the peaks at 960 (CH out-of-plain/trans), 833, 697, and 609 cm^−1^ *v*(C-Cl) [19].

**Figure 7:**
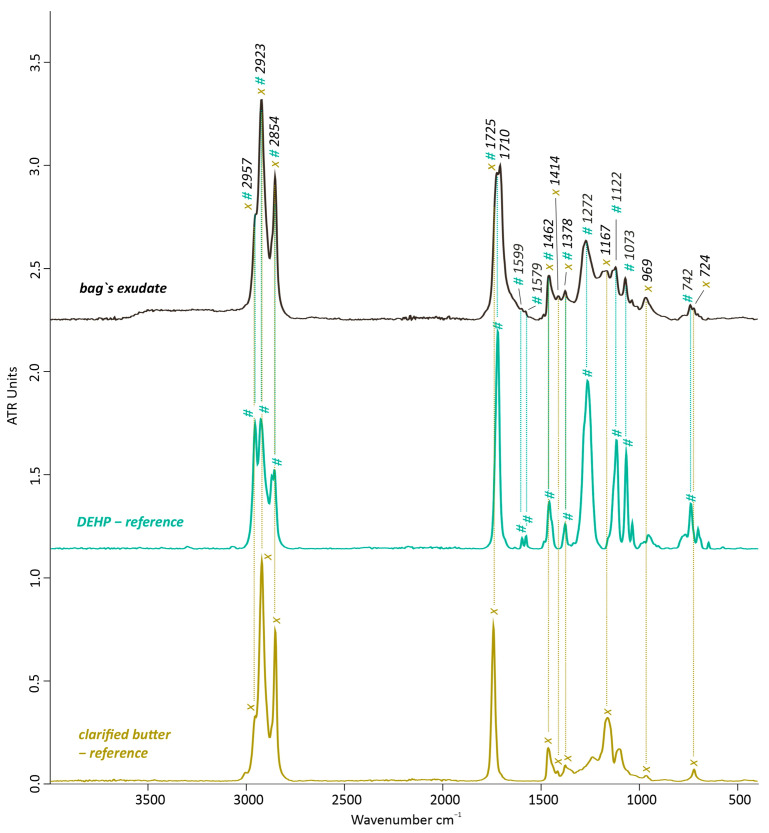
FTIR-ATR spectrum of the sticky exudates from the filled bag’s surface compared with DEHP (bis(2-ethylhexyl) phthalate) and clarified butter as reference spectra. # indicates the contribution bands of DEHP and x of clarified butter.

Differently from the transparent non-sticky bags, the exudate from the bag’s surface (taken from measuring point III., Figure 2) was mostly characterized by the presence of fatty organic compounds and, to a lesser extent, by phthalate-based plasticizers, suggesting their migration and diffusion towards the surface. The typical absorption bands for fats and oils—also shown by the IR spectrum of the clarified butter as reference material in Figure 7—include *v*_a_(C-H) in CH_3_ at 2957 cm^−1^, *v*_a_(C-H) at 2923, and *v*_s_(C-H) at 2854 cm^−1^ in CH_2_ of the aliphatic hydrocarbon chain of the fatty acids and their terminal ends, but also the doublets at 1725 and 1710 cm^−1^ of *v*(C=O) in esters and carboxyl acids, respectively, which highly overlapped the bands of the phthalate-based plasticizers at similar wavenumbers.

Additionally, the peak at 1462 cm^−1^ is associated with *δ*(CH) of CH_2_ and CH_3_ groups—overlying with the *v*(C-O-C) in the phthalate-based plasticizer—while the less intense peak at 1414 cm^−1^ is due to *ω*(CH_2_) and the one at 1378 cm^−1^ of *ω*(CH2) in the organic fat components and of *δ*(CH_3_) in the phthalate-based plasticizers. The triglycerides ester linkage can be seen as *v*(C-O) at 1167 cm^−1^, a position band for which *δ*(CH) also contributes. The peak at 724 cm^−1^ is a slight overlaid of CH_2_ rocking vibrations and *cis* –C=(C-H) out-of-plane deformations [37], while non-conjugated *trans* (C-H) deformations are at 969 cm^−1^. On the other hand, a minor contribution in the IR spectrum (Figure 7) is represented by phthalate-based plasticizer peaks such as those of DEHP at 1272 and 1122 cm^−1^ (CH aromatic in-plane deformation) and at 1073 cm^−1^ (CH or C-C aromatic in-plane deformation). Slightly noticeable is the small doublet at 1599 and 1579 cm^−1^ referring to the characteristic *v*(C=C) in phthalic compounds [3,13,18,19,33,34] and a minor peak at 742 cm^−1^ of the *δ*(=C-H) out-of-plain of a 1, 2 disubstituted benzene ring.

**Figure 8:**
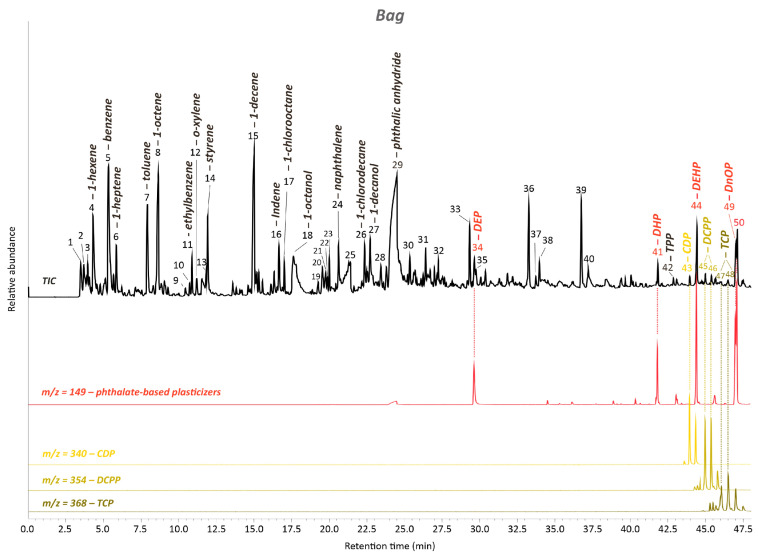
Total ion chromatogram (TIC) of the transparent bag’s sample acquired by Py-GC/MS. The numbered peaks of the TICs are listed and described in Table S2. DEP: diethyl phthalate; DHP: dihexyl phthalate; DEHP: bis(2-ethylhexyl) phthalate; DnOP: di-n-octyl phathalate; TPP: triphenyl phosphate Extracted ion chromatograms (EICs) of the ion mass with *m*/*z* = 149 for phthalate-based plasticizers, *m*/*z* = 368 and 354 and 340 for cresyl diphenyl phosphate (CDP), dicresyl phenyl phosphate (DCPP), and isomers of tricresyl phosphate (TCP), respectively, are characteristic for the group of phenyl phosphate plasticizers/flame retardants.

The FTIR-ATR obtained results for the transparent bag’s sample were again confirmed by the Py-GC/MS analysis, which allowed us to add further information. In the pyrogram of the transparent non-sticky bags, most characteristic pyrolysis products of PVC [35,36] are noticeable within the retention times (RT) lower than 23 min (peaks #5,7,11,12,14,16,24; Figure 8, Table S2). While the exact identification of phthalate-based plasticizers is challenging with FTIR-ATR because of their similar molecular bond vibrations, Py-GC/MS allows their correct characterization. Next to phthalic anhydride (#29), DEHP can be confirmed by peak #44 as the most abundant type of phthalate-based plasticizer in the sample analyzed (Figure 8, Table S2). While the most intense ion at 149 in the mass-to-charge ratio *(m*/*z)* can be seen as general confirmation for phthalates, 167 and 279 can be considered confirmation ions of DEHP [27,28,38]. Other phthalic compounds, such as diethyl phthalate (DEP) (#34), dihexyl phthalate (DHP) (#41), di-n-octyl phthalate (DnOP) (#49), and phthalic acid decyl hexyl ester (#50), could be found.

Together with TPP (#42), some traces of TCP, CDP, and DCPP could be detected as well in the form of different pyrolysis products (#47,48, #43, and #45,46, respectively) (Figure 8, Table S2), similar to the Py-GC/MS results obtained for the cover plate sample.

### 3.2. Replica Materials

#### 3.2.1. JEDI Plate

**Figure 9:**
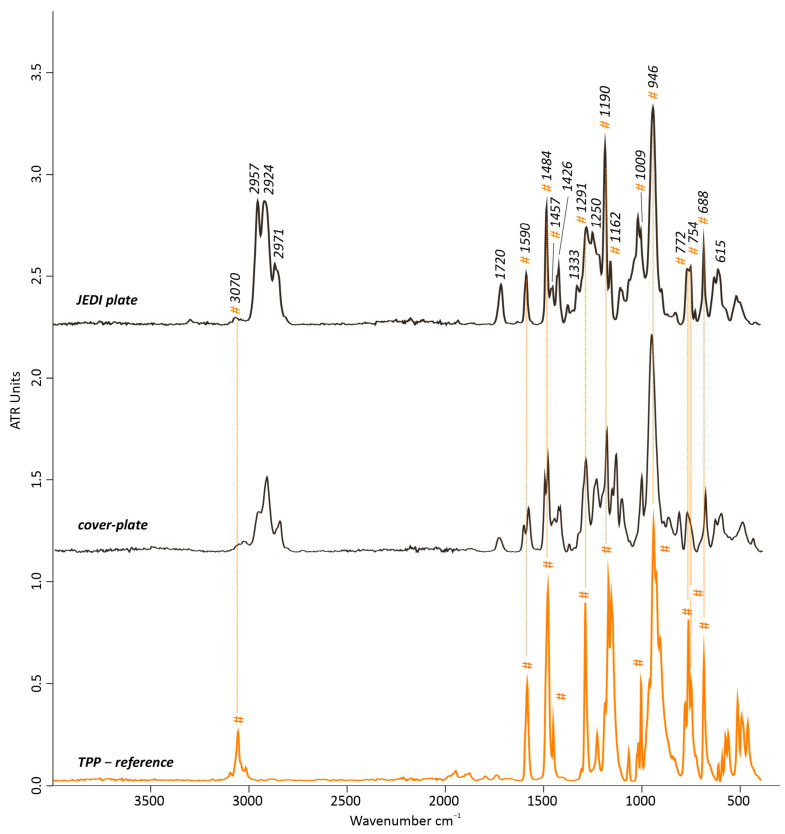
FTIR-ATR spectrum of the JEDI plate’s sample compared with the artwork cover plate and TPP (triphenyl phosphate) as a reference spectrum. # indicates the contribution bands of TPP.

The material composition of the JEDI plate was partly similar to that of the cover plate of Roth artwork. The FTIR-ATR results of the JEDI plate showed a higher similarity with the spectrum of TPP than to the TCP found in the cover plate, in addition to some of the PVC bands (Figure 9). The TPP bands were identified at 3070 cm^−1^ (aromatic *v*(C-H)), 1590, 1484, and 1457 cm^−1^ (aryl substituents and aryl CH_3_ vibrations), 1291 cm^−1^ *v*(P-O), 1190 and 1162 cm^−1^ (aryls in plane C-H), 1009 cm^−1^ *v*(P-O), 946 cm^−1^ (overlapping of the aryl vibrations of the PO [32] with the *δ*(C-H) out-of-plane in PVC [3,19], 772, 754, and 688 cm^1^ (aromatic *δ*(C-H)). A minor contribution of the PVC bands corresponded at 2964 *v*_a_(C-H) in CH_3_, 2924 *v*_a_(C-H), and 2867 cm^−1^ *v*_s_(C-H) in CH_2,_ 1426 (*δ*(CH-Cl)), 1333 cm^−1^ (*w*(C-H)), and 615 cm^−1^ (*v*(C-Cl)).

**Figure 10:**
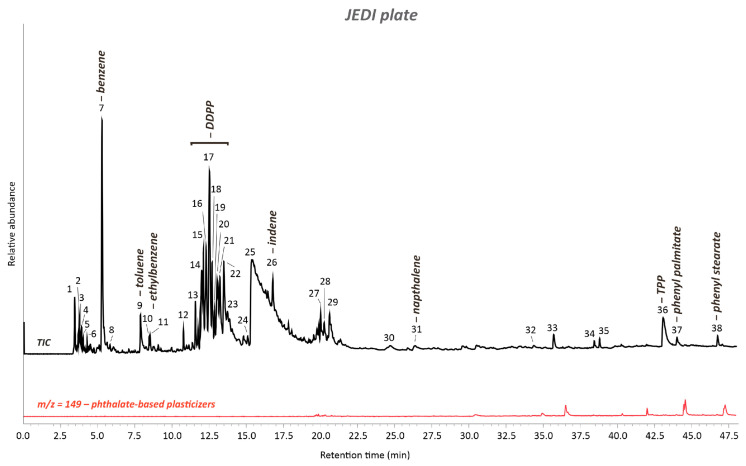
Total ion chromatogram (TIC) of the JEDI plate’s sample acquired by Py-GC/MS. The numbered peaks of the TICs are listed and described in Table S3. DDPP: di-isodecyl phenyl phospite. TPP: triphenyl phosphate. Extracted ion chromatogram (EIC) of the ion mass with *m*/*z* = 149 for phthalate-based plasticizers showing their absence in the pyrogram.

Moreover, the Py-GC/MS pyrogram shed some light on the typology of additives used for the JEDI plate, pointing not only to the presence of TPP (peak #36) as a plasticizer/flame retardant but also to the presence of di-isodecyl phenyl phosphite (DDPP) as an antioxidant (peaks #13–23), phenyl palmitate (peak #37), and phenyl stearate (peak #38) as bio-based chemical additives (Figure 10, Table S3). In contrast to the artwork cover plate, no significant pyrolysis products of phthalate-based plasticizers were evidenced in the EIC at *m*/*z* = 149 (Figure 10).

#### 3.2.2. Dia-Bag

**Figure 11:**
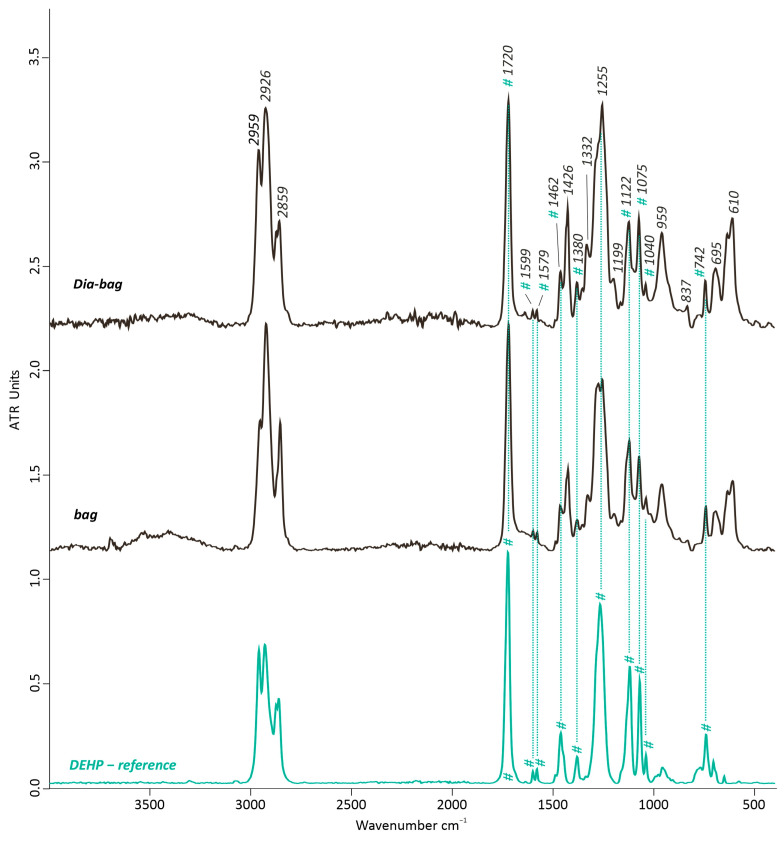
FTIR-ATR spectrum of the Dia-bag’s sample compared with the artwork’s bag and DEHP (bis(2-ethylhexyl) phthalate) as reference spectrum. # indicates the contribution bands for DEHP.

Similar to the FTIR-ATR results of the cover plate and JEDI plate, an almost identical spectrum of the Dia bag to those of the artwork’s bag was gathered (Figure 11), which suggested first that the DEHP plasticizer was included in the formulation as the main additive. Indeed, several representative IR bands of DEHP—also previously described (see Section 3.1.2)—were detected at 1720 cm^−1^ *v*(C=O), 1599 and 1579 cm^−1^ *v*(C=C), 1462 cm^−1^ *v*(C-O-C), 1380 cm^−1^ *δ*(CH_3_), 1122 cm^−1^ (CH aromatic in-plane deformation), 1075 cm^−1^ (CH or C-C aromatic in-plane deformation), 1040 cm^−1^ (*v*(C-O)), and at 742 cm^−1^ (*δ*(=C-H)). Some bands of PVC were revealed at 2959 *v*_a_(C-H) in CH_3_, 2926 *v*_a_(C-H) and 2859 cm^−1^ *v*_s_(C-H) in CH_2_, 1426 cm^−1^ (*δ*(CH-Cl), 1332 cm^−1^ *w*(C-H), 1255 cm^−1^ *δ*CH-Cl out-of-plain, 1199 cm^−1^ *ω*(CH), 959 cm−^1^ CH out-of-plain/trans, 695 cm^−1^, and 610 cm^−1^ *v*(C-Cl).

**Figure 12:**
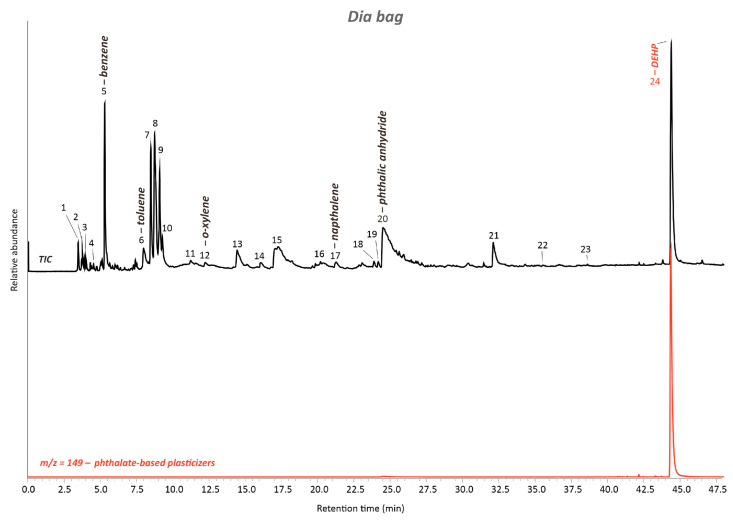
Total ion chromatogram (TIC) of the Dia-bag’s sample acquired by Py-GC/MS. The numbered peaks of the TICs are listed and described in Table S4. Extracted ion chromatogram (EIC) of the ion mass with *m*/*z* = 149 for phthalate-based plasticizers. DEHP: bis(2-ethylhexyl) phthalate.

In addition to detecting the fundamental peaks of PVC, Py-GC/MS analysis confirmed the DEHP evidence. As shown in Figure 12 and described in Table S4, typical pyrolysis products of DEHP were registered in the TIC and corresponded to 2-ethylhexane (#7), 3-ethyl-3-hexene (#8), 2-heptene, 3-methyl- (#9), 2-ethylhexanol (#15), phthalic anhydride (#20), 2-ethylhexyl benzoate (#21), and phthalic acid, bis(2-ethylhexyl) ester (DEHP) (#22) [27,28,38]. The other additives found in the artwork’s bag, such as TPP + CDP + DEP + DHP + DEHP + DnOP + phthalic acid decyl hexyl ester, were not part of the Dia-bags. This highlights once more the differences in chemical composition between the p-PVC of the investigated artwork from 1969 and the p-PVC of the Dia-bags of the last years and the difficulties in having full reproducible replicas for the test series.

### 3.3. Artificial Thermal Aging Studies

#### 3.3.1. Dia-Bag without Filling and JEDI Plate

**Figure 13:**
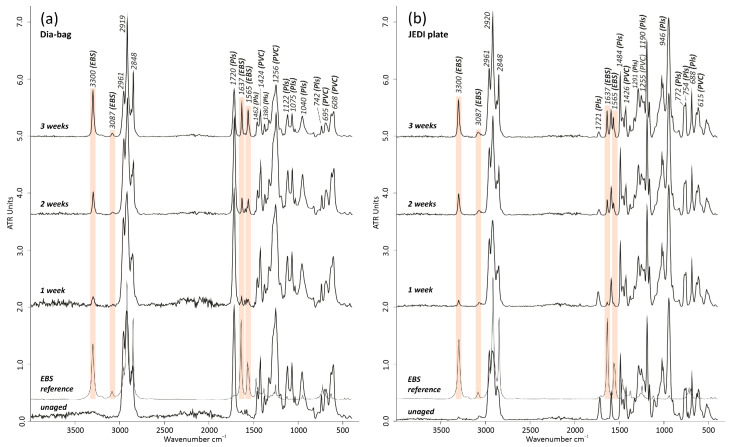
FTIR-ATR spectra comparison of the unaged and artificially thermally aged (**a**) Dia-bags and (**b**) JEDI plate at 70 °C and for 1, 2, and 3 weeks, both compared with the EBS (N,N’-ethylenebis(stearamide)) reference IR spectrum. The light pink color highlights the formation of some IR bands during the aging, corresponding to the EBS contribution, particularly pronounced after 3 weeks of thermal aging. Pls: plasticizer; PVC: polyvinyl chloride.

The Dia-bags without filling and JEDI plates, considered replica materials for this research, were first artificially thermally aged to study the thermal decomposition of both types of PVC. No signs of stickiness were observed on the surface, but only a change in color. The Dia-bags optical changes became quickly apparent during the thermal aging, shifting their transparency to a yellowish and a reddish hue after 21 days at 70 °C (Figure S1). Additionally, a slight shrinkage of the samples as well as an increase in embrittlement could be recognized. Despite those optical hints towards dehydrochlorination and a loss of plasticizers, neither the accumulation of liquids on the sample’s surface nor a change in the bands associated with the plasticizers could be detected. The FTIR-ATR spectra of the thermally aged Dia-bags and JEDI plate showed no significant variations in the bands of the plasticizers (Figure 13a,b). Only a slight relative intensity decrease, particularly of the band at ~1720 cm^−1^ from the Dia-bag and in a more accentuated way from the JEDI plate, pointed towards a loss of plasticizers [3,13,17,18]. Regarding the PVC bands, a significant decrease in their relative intensity at 1424 δ(CH-Cl), 1256 δCH-Cl o-o-p, 695, and 608 cm^−1^ *v*(C-Cl) may suggest a dehydrochlorination process that occurred progressively after 3 weeks of thermal aging at 70 °C. This effect was mostly observed in the Dia-bags, in contrast to the JEDI plate.

Despite this, all samples—Dia bags and JEDI plates—showed the formation and progressive increase of four main bands at 3300, 3087, 1637, and 1565 cm^−1^, complemented by an increase and sharpening of the peaks at ~2919 and ~2848 cm^−1^ (Figure 13a,b). Only one text could be found that describes the formation of these peaks in naturally aged p-PVC samples, which were additionally thermally aged at cycles of 80 °C for two days and 25 °C for one day with 65% RH [14]. Royaux et al. [14] described those peaks in p-PVC samples as possibly related to amino-based additives, likely zein, used to make the material more hydrophobic and for protection against mold growth. Nevertheless, a quite close look into books about PVC additives instead raises the suggestion that an amino-wax like N,N’-ethylenebis(stearamide) (EBS) seems a more appropriate correlation, which is used as a slip agent and lubricant in PVC formulations [39]. The comparison of the FTIR-ATR spectra between this type of additive (Hummel Industrial Polymers 3.S01 Library) and the bands found in the thermally aged samples also highly supports this assumption (Figure 10).

In this case, the peaks at 3300 cm^−1^ can be assigned to *v*(N-H), 3087 cm^−1^ to an overtone of δ(N-H), and 1565 cm^−1^ to in-plane δ(N-H), while the absorption band at 1640 cm^−1^ corresponds to *v*(C=O), originating from the amino group (-CONH) of the EBS. The increase of the peaks at 2919 and 2859 cm^−1^ is attributed to the aliphatic *v*(C-H) of the EBS [40,41,42]. Additionally, it has been reported elsewhere [43,44] that hexadecanenitrile and octadecanenitrile are produced during GC/MS and Py-GC/MS analyses of EBS. These two compounds were then detected in a very tiny amount in the unaged Dia-bags by Py-GC/MS (Figure 11, peak #22, 23) but not in the JEDI plate, likely because of their tiny percentage content. Since EBS can also have negative effects on the stability of the PVC, only minor amounts of EBS are used, especially in combination with tin stabilizers, and moreover, in the US and Asia rather than actually in Europe [31].

#### 3.3.2. Thermal Aging on Dia-Bags with Fillings

**Figure 14:**
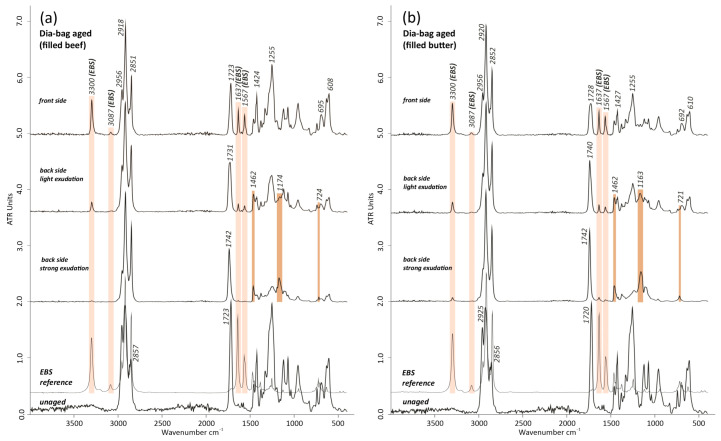
FTIR-ATR spectra comparison of the artificial thermally aged Dia-bags (**a**) filled with beef, including front side, back side characterized by slight and strong exudation, and unaged Dia-bags, and (**b**) filled with clarified butter, including front side, back side characterized by slight and strong exudation, and unaged Dia-bags, both compared with the EBS (N,N’-ethylenebis(stearamide)) reference IR spectrum. The light pink color highlights the formation of some IR bands of EBS during the aging, particularly pronounced in the front side, while the IR bands in brown color indicate fatty organic component-based exudate mostly in the back side of the aged Dia-bags.

Since the effects of the bag’s filling were of major interest for the present case study, artificial thermal aging tests of the Dia-bags filled with either minced meat or clarified butter were additionally carried out. By optically observing the thermally aged bags with fillings, it could be seen that a slight shift in color occurred, but no stickiness was present on the front sides, which was, on the other hand, noticed on the back sides. By comparing the FTIR-ATR results obtained from the non-sticky front side of the aged Dia-bags and the sticky back side, filled with beef or clarified butter, some differences were observed. As shown in Figure 14a,b, the front side of the Dia-bags was characterized by the presence of EBS, according to the four main intense peaks at 3300 cm^−1^ *v*(N-H), 3087 and 1567 cm^−1^ *v*(N-H), and 1635 cm^−1^ *v*(C=O), which were similarly identified in the aged Dia-bags without fillings (Section 3.3.1). While those peaks were highly noticeable in the IR spectrum acquired on the front side, they became less detectable in the IR spectra of the back side in correspondence to small but also strong exudates, which preferably diffused/migrated toward those sides. They consisted mostly of IR bands of fatty components at 1462 cm^−1^ δ(CH), 1172 cm^−1^ *v*(C-O), and at 724 cm^−1^ *cis* –C=(C-H), with a shifted *v*(C=O) band to higher wavenumbers (from ~1723 to ~1742 cm^−1^) of the ester groups, similarly to those detected on the exudate’s bags of the artwork (see Section 3.1.2). This can point to the fact that the fatty organic components exudate/migrate in a different direction, since fatty exudates were mostly found on the bags on the front side of the artwork. This can be influenced or played by different factors, such as whether the bags are more or less exposed to air/oxygen, the temperature at the surface, and whether they are vertically or horizontally positioned.

The small doublet at 1599 and 1579 cm^−1^ referring to phthalates-based plasticizers is still detectable in the thermally aged replicas (front side and back side, light exudation), although with a lower relative intensity and partially overlapped by the EBS bands (Figure 14a,b).

## 4. Conclusions

This research provides useful information about the main organic constituents, their state of degradation, and their distribution into the p-PVC-based materials as part of the *PO^E^METRIE* artwork (1968) by Dieter Roth, which can guide further studies on the other copies of the edition *PO^E^METRIE* but also on other types of p-PVC-based modern-contemporary art objects. Hereafter, the following major conclusions can be highlighted:

− It was important to notice that the distribution of dust and particle agglomeration, as well as sticky areas, were found mostly in some specific areas of the artwork by UV/Vis imaging, which suggested a vertically aligned presentation or hanging of the object, which was not clear and known from the historical documentation. Also, while the yellowish fluorescence in the UV images points toward organic fatty compounds, the pH measurements helped to characterize the exudates as highly acidic.
− Analytical investigations allowed the determination of the exact chemical composition of the p-PVC-based cover plate and bags, as well as the replicas selected for the thermal aging studies, additionally pointing out their differences in terms of plasticizer types. Despite the extensive agreement between the FTIR-ATR spectra of the samples from the artwork and the selected replica materials, Py-GC/MS showed clear differences in the material composition.
− Very importantly, this research also demonstrated that despite several types of PVC-based materials that can be characterized by different plasticizer-type compositions, they still may present similar degradation pathways, such as migration of the plasticizers, a decrease in flexibility and an increase in brittleness, as well as an increase in yellowing as a color change. For instance, the p-PVC-based cover plate and bags of the artwork and the recent p-PVC-based replicas used for the thermal aging studies were all affected by those degradation pathways, regardless of their slightly different plasticizer compositions as determined by Py-GC/MS.
− On the other hand, the well-known stickiness issue can be strictly influenced by the intrinsic characteristics of the p-PVC material formulation, such as plasticizer composition, but also by the environmental conditions. Indeed, the most recent p-PVC formulation, such as the one used for this study, was not affected by stickiness after artificially accelerated thermal aging. Instead, the new typology of p-PVC representing the replicas suffered from a migration of a detected ammine-wax type of lubricant, which for the first time was successfully identified as N,N’-ethylenebis(stearamide) (EBS) by FTIR-ATR and only after the thermal aging. These results open new aspects and degradation pathways of the most recent p-PVC formulation, which need to be considered not only in the field of modern and contemporary art but, for instance, in industrial applications and food packaging, and which provide an important source of information.
− The determination of fatty organic components mostly on the back side of the thermally aged replica’s bags filled with minced meat and clarified butter points to the fact that they can follow different directions during exudation or migration, since fatty exudates were mostly found on the front side of the artwork. Factors such as air/oxygen exposure, temperature at the surface, plasticizers, and additive types, whether vertical or horizontal, should be considered. This can thus significantly suggest that special care should be considered while planning the exhibition of those artworks and in which position they should be hung and placed.

Further studies, possibly based on quantitative FTIR investigations and considering indoor conditions, for instance, to determine the competition between the yellowing effect and the migration and accumulation of the plasticizers on the surface with consequent stickiness issues, would be beneficial and relevant for the art and conservation context and for polymer research areas.

Finally, this work provides for the first time valuable scientific information not only about one of Dieter Roth’s artworks but also on plasticizer migration and fat diffusion in p-PVC. The results here gained may be of help for possible future research studies on further famous masterpieces by Dieter Roth and beyond.

## Figures and Tables

**Table 1:** Summary of the different examination spots on the artwork, the respective measuring methods employed, and the results obtained. PVC: polyvinyl chloride; TCP: tricresyl phosphate; TPP: triphenyl phosphate; CDP: cresyl diphenyl phosphate; DCPP: dicresyl phenyl phosphate; DEP: diethyl phthalate; DIBP: diisobutyl phthalate; DBP: dibutyl phthalate; DHP: dihexyl phthalate; DEHP: bis(2-ethylhexyl) phthalate.

Spot #	Material	Method	Results
I.	Plate (non-sticky)	FTIR-ATR	PVC + TCP
		Py-GC/MS	PVC + TCP + TPP + CDP + DCPP + DEP + DIBP + DBP + DHP + DEHP + benzenepropanoyl bromide
I.i		pH	4.9
I.ii		pH	4.9
I.iii	Plate’s exudates	FTIR-ATR	TCP
I.iv	Plate (sticky area)	pH	4.6
II.	Bag (non-sticky)	FTIR-ATR	PVC + DEHP
		Py-GC/MS	PVC + TPP + CDP + DEP + DHP + DEHP + DnOP + phthalic acid decyl hexyl ester
II.i		pH	5.8
II.ii		pH	5.1
III.	Bag’s exudates	FTIR-ATR	DEHP + fats
III.i	Bag (sticky area—directly on a droplet)	pH	3.9
III.ii		pH	4.5
III.iii		pH	4.6

## Data Availability

The data presented in this study are available on request from the corresponding author.

## Supplementary Materials

The following supporting information can be downloaded at: https://www.mdpi.com/article/10.3390/polym15234558/s1, Figure S1: Gradual color change observed in the Dia-bags from ungaged to two weeks and three weeks of thermal aging at 70 °C; Table S1: Main pyrolysis products obtained by Py-GC/MS of the investigated artwork’s cover plate at their corresponding retention time (RT min) and mass to charge ratio (*m*/*z*) (base peak marked in bold); Table S2: Main pyrolysis products obtained by Py-GC/MS of the investigated artwork’s bag at their corresponding retention time (RT min) and mass to charge ratio (*m*/*z*) (base peak marked in bold); Table S3: Main pyrolysis products obtained by Py-GC/MS of the investigated JEDI plate at their corresponding retention time (RT min) and mass to charge ratio (*m*/*z*) (base peak marked in bold); Table S4: Main pyrolysis products obtained by Py-GC/MS of the investigated Dia-bag at their corresponding retention time (RT min) and mass to charge ratio (*m*/*z*) (base peak marked in bold).
