# Supplementary material for: Towards the Understanding of the Aging Behavior of p-PVC in Close Contact with Minced Meat in the Artwork POEMETRIE by Dieter Roth
Source: Polymers (Basel). 2023 Nov 28;15(23):4558. doi: 10.3390/polym15234558 (PMC10707740; doi:10.3390/polym15234558)
Supplement: Supplementary file 1 [file polymers-15-04558-s001.zip › polymers-2671120-supplementary.pdf]

*Supplementary Materials*

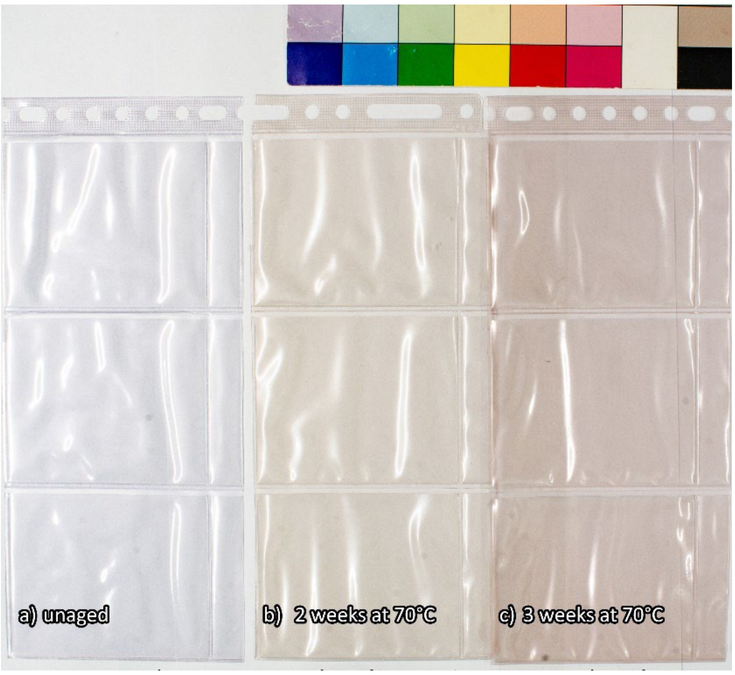


**Figure S1:** Gradual colour change observed in the Dia-bags from ungaged to two weeks and three weeks of thermal aging at 70 °C

| Peak # | RT min | Compound Name | m/z |
|---|---|---|---|
| 1 | 3.52 | Butadiene | 54 |
| 2 | 3.74 | 1-Pentene | **55**, 70 |
| 3 | 3.97 | 1,3-Cyclopentadiene | 66 |
| 4 | 4.33 | 1-Hexene | **56**, 69, 84 |
| 5 | 5.33 | Benzene | 51, **78** |
| 6 | 5.87 | 1-Heptene | 55, **56**, 70, 83, 98 |
| 7 | 7.92 | Toluene | 51, 65, 74, 89, **91** |
| 8 | 8.57 | 1-Octene | **55**, 57, 70, 83, 97, 112, 113 |
| 9 | 10.44 | Chlorobenzene | 51, 77, **112** |
| 10 | 10.88 | Ethylbenzene | 51, 65, 77, **91**, 103, 106 |
| 11 | 11.20 | o-Xylene | 51, 65, 77, **91**, 103, 106 |
| 12 | 11.81 | 1-Nonene | **56**, 69, 83, 97, 111, 126 |
| 13 | 11.92 | Styrene / Benzenepropanoyl bromide | 51, 78, **104** |
| 14 | 14.92 | 1-Decene | **55**, 70, 83, 97, 111, 140 |
| 15 | 16.65 | Indene | 50, 63, 74, 76, 89, 109, **116** |
| 16 | 18.07 | o-Cresol | 51, 77, 79, 107, **108** |
| 17 | 19.25 | Benzene, 1-butenyl | 51, 65, 77, 91, 105, **117**, 132 |
| 18 | 19.53 | 1H-Indene, 3-methyl- | 51, 64, 77, 91, 105, 115, 128, **130** |
| 19 | 19.75 | 1H-Indene, 1-methyl- | 51, 64, 77, 91, 105, 115, 128, **130** |
| 20 | 19.86 | Naphthalene, 1,2,3,4-tetrahydro- | 51, 65, 77, 91, **104**, 115, 128, 132 |
| 21 | 19.99 | Naphthalene, 1,4-dihydro- | 51, 64, 77, 89, 102, 115, 128, **130** |
| 22 | 20.61 | Naphthalene | 51, 63, 75, 102, **128** |
| 23 | 22.14 | Undecane, 2,4-dimethyl- | **51**, 71,85, 99, 113, 126, 141, 155, 169, 184 |
| 24 | 22.47 | Heptadecane | **57,** 71, 85, 99, 113, 127, 141, 155, 169, 183, 197, 240 |
| 25 | 23.42 | Naphthalene, 1-methyl- | 57, 63, 71, 87, 89, 115, 138, **142** |
| 26 | 24.47 | Phthalic anhydride | 50, **76**, 104, 148, 149 |
| 27 | 25.27 | Diphenyl | 76, 115, **154** |
| 28 | 25.34 | Tetradecane | **57**, 71, 85, 99, 113, 127, 141, 155, 169, 198 |
| 29 | 27.23 | Heptadecane | **57**, 71, 85, 99, 113, 127, 141, 155, 169, 183, 197, 240 |
| 30 | 29.62 | Diethyl Phthalate (DEP) | 65, 76, 93, 105, 121, **149,** 177, 222 |
| 31 | 29.74 | Fluorene | 63, 82, 115, 139, **166** |
| 32 | 31.39 | Heneicosane | **57**, 71, 85, 99, 113, 127, 141, 155, 169, 183, 197, 211, 225, 296 |
| 33 | 33.24 | Benzoic acid, octyl ester | 70, 77, 97, 105, **123**, 135, 163 |
| 34 | 33.71 | Anthracene | 51, 63, 76, 89, 115, 139, 152, 163, **178** |
| 35 | 34.49 | Diisobutyl phthalate (DIBP) | 57, 76, 93, 104, 121, **149**, 167, 205, 223 |
| 36 | 36.12 | Bibutyl phthalate (DBP) | 76, 104, **149**, 205, 223, 278 |
| 37 | 36.19 | Palmitic acid | 60, **73**, 83, 129, 143, 171, 185, 199, 241, 284 |
| 38 | 36.69 | Benzoic acid, octyl ester | **56,** 77, 83, 105, 123, 163 |
| 39 | 39.28 | Stearic acid | 60, **73**, 83, 101, 129, 143, 157, 171, 185, 213, 227, 256 |
| 40 | 41.80 | Dihexyl phthalate (DHP) | 55, 76, 85, 104, 121, 132, **149**, 167, 233, 251 |
| 41 | 42.85 | Triphenyl phosphate (TPP) | 51, 65, 77, 94, 115, 141, 169, 215, 233, 249, **326** |
| 42 | 43.94 | Cresyl diphenyl phosphate (CDP) | 65, 77, 108, 115, 152, 165, 184, 229, 247, 263, **340** |
| 43 | 44.35 | Cresyl diphenyl phosphate (CDP) | 65, 77, 108, 115, 152, 165, 184, 229, 247, 263, **340** |
| 45 | 44.48 | Bis(2-ethylhexyl)phthalate (DEHP) | 57, 70, 83, 104, 121, **149**, 167, 233, 251, 279 |
| 45 | 44.04 | Dicresyl phenyl phosphate (DCPP) | 65, 77, 91, 153, 165, 198, 229, 247, 261, **354**, 355 |
| 46 | 45.54 | Dicresyl phenyl phosphate (DCPP) | 65, 77, 91, 153, 165, 198, 229, 247, 261, **354**, 355 |
| 47 | 46.18 | Tri-m-cresyl phosphate (TCP) | 51, 65, 77, 91, 108, 165, 179, 195, 211, 243, 261, 277, 291, 354, **368** |
| 48 | 46.87 | Tri-m-cresyl phosphate (TCP) | 51, 65, 77, 91, 108, 165, 179, 195, 211, 243, 261, 277, 291, 354, **368** |
| 49 | 47.41 | Tri-m-cresyl phosphate (TCP) | 51, 65, 77, 91, 108, 165, 179, 195, 211, 243, 261, 277, 291, 354, **368** |
| 50 | 47.96 | Tri-m-cresyl phosphate (TCP) | 51, 65, 77, 91, 108, 165, 179, 195, 211, 243, 261, 277, 291, 354, **368** |

**Table S1**: Main pyrolysis products obtained by Py-GC/MS of the investigated **artwork's cover-plate** at their corresponding retention time (RT min) and mass to charge ratio (*m*/*z*) (base peak marked in bold).

| Peak # | RT min | Compound Name | m/z |
|---|---|---|---|
| 1 | 3.52 | Butadiene | 54 |
| 2 | 3.68 | 1-Pentene | **55**, 70 |
| 3 | 3.98 | 1,3-Cyclopentadiene | 66 |
| 4 | 4.29 | 1-Hexene | **56**, 69, 84 |
| 5 | 5.31 | Benzene | 51, **78** |
| 6 | 5.84 | 1-Heptene | 55, **56**, 70, 83, 98 |
| 7 | 7.90 | Toluene | 51, 65, 74, 89, **91** |
| 8 | 8.63 | 1-Octene | **55**, 57, 70, 83, 97, 112, 113 |
| 9 | 10.43 | Chlorobenzene | 51, 77, **112** |
| 10 | 10.71 | 1-Chlorohexane | 55, 69, 71, 84, **91**, 93 |
| 11 | 10.86 | Ethylbenzene | 51, 65, 77, **91**, 103, 106 |
| 12 | 11.78 | o-Xylene | 51, 65, 77, **91**, 103, 106 |
| 13 | 11.81 | 1-Nonene | **56**, 69, 83, 97, 111, 126 |
| 14 | 11.91 | Styrene | 51, 78, **104** |
| 15 | 14.99 | 1-Decene | **55**, 70, 83, 97, 111, 140 |
| 16 | 16.44 | Indene | 50, 63, 74, 76, 89, 109, **116** |
| 17 | 17.03 | 1-Chlorooctane | 55, 57, 69, 83, **91**, 105, 119 |
| 18 | 17.61 | 1-Octanol | **55**, 56, 70, 84, 95, 97, 112 |
| 19 | 19.25 | Benzene, 1-butenyl | 51, 65, 77, 91, 105, **117**, 132 |
| 20 | 19.54 | 1H-Indene, 3-methyl- | 51, 64, 77, 91, 105, 115, 128, **130** |
| 21 | 19.75 | 1H-Indene, 1-methyl- | 51, 64, 77, 91, 105, 115, 128, **130** |
| 22 | 19.86 | Naphthalene, 1,2,3,4-tetrahydro- | 51, 65, 77, 91, **104**, 115, 128, 132 |
| 23 | 19.99 | Naphthalene, 1,4-dihydro- | 51, 64, 77, 89, 102, 115, 128, **130** |
| 24 | 20.61 | Naphthalene | 51, 63, 75, 102, **128** |
| 25 | 21.40 | Benzenecarboxylic acid | 51, 77, **105**, 122 |
| 26 | 22.34 | 1-Chlorodecane | 55, 57, 69, 83, **91**, 105, 107, 119 |
| 27 | 22.70 | 1-Decanol | **55**, 70, 97, 112, 129, 140 |
| 28 | 23.42 | Naphthalene, 1-methyl- | 57, 63, 71, 87, 89, 115, 138, **142** |
| 29 | 24.47 | Phthalic anhydride | 50, **76**, 104, 148, 149 |
| 30 | 25.34 | Diphenyl | 76, 115, **154** |
| 31 | 26.39 | Hexane, 1,1'-[methylenebis(oxy)]bis- | 57, 71, 83, **85**, 96, 115 |
| 32 | 27.23 | Heptadecane | **57**, 71, 85, 99, 113, 127, 141, 155, 169, 183, 197, 240 |
| 33 | 29.33 | Benzoic acid, hexyl ester | **56,** 69, 77, 85, 105, 123, 135 |
| 34 | 29.62 | Diethyl Phthalate (DEP) | 65, 76, 93, 105, 121, **149,** 177, 222 |
| 35 | 29.74 | Fluorene | 63, 82, 115, 139, **166** |
| 36 | 33.24 | Benzoic acid, octyl ester | 70, 77, 97, 105, **123**, 135, 163 |
| 37 | 33.71 | Anthracene | 51, 63, 76, 89, 115, 139, 152, 163, **178** |
| 38 | 33.95 | Octane, 1,1'-oxybis- | **57,** 71, 84, 97, 111, 129, 143 |
| 39 | 36.74 | Benzoic acid, octyl ester | **56,** 77, 83, 105, 123, 163 |
| 40 | 37.20 | Tritetracontane | **57**, 71, 85, 97, 111, 127, 141, 155, 169, 197, 239 |
| 41 | 41.82 | Dihexyl phthalate (DHP) | 55, 76, 85, 104, 121, 132, **149**, 167, 233, 251 |
| 42 | 42.85 | Triphenyl phosphate (TPP) | 51, 65, 77, 94, 115, 141, 169, 215, 233, 249, **326** |
| 43 | 43.94 | Cresyl diphenyl phosphate (CDP) | 65, 77, 108, 115, 152, 165, 184, 229, 247, 263, **340** |
| 44 | 44.42 | Bis(2-ethylhexyl)phthalate (DEHP) | 57, 70, 83, 104, 121, **149**, 167, 233, 251, 279 |
| 45 | 44.97 | Dicresyl phenyl phosphate (DCPP) | 65, 77, 91, 153, 165, 198, 229, 247, 261, **354**, 355 |
| 46 | 45.37 | Dicresyl phenyl phosphate (DCPP) | 65, 77, 91, 153, 165, 198, 229, 247, 261, **354**, 355 |
| 47 | 46.02 | Tri-m-cresyl phosphate (TCP) | 51, 65, 77, 91, 108, 165, 179, 195, 211, 243, 261, 277, 291, 354, **368** |
| 48 | 46.48 | Tri-m-cresyl phosphate (TCP) | 51, 65, 77, 91, 108, 165, 179, 195, 211, 243, 261, 277, 291, 354, **368** |
| 49 | 46.99 | Di-n-octyl phthalate (DnOP) | 57, 104, **149**, 167, 279 |
| 50 | 47.09 | Phthalic acid, decyl hexyl ester | 57, 85, **149,** 167, 251, 307 |

**Table S2**: Main pyrolysis products obtained by Py-GC/MS of the investigated **artwork's bag** at their corresponding retention time (RT min) and mass to charge ratio (*m*/*z*) (base peak marked in bold).

| Peak # | RT min | Compound Name | m/z |
|---|---|---|---|
| 1 | 3.52 | Butadiene | 54 |
| 2 | 3.71 | Isoprene | 53, **67** |
| 3 | 3.81 | 1,3-Pentadiene, (E)- | 53, **67** |
| 4 | 3.88 | 1,3-Cyclopentadiene | 66 |
| 5 | 3.98 | 4-Penten-1-ol | 53, 56, **67**, 85 |
| 6 | 4.24 | 1-Hexene | **56**, 69, 84 |
| 7 | 5.25 | Benzene | 51, **78** |
| 8 | 5.79 | 1-Heptene | 55, **56**, 70, 83, 98 |
| 9 | 7.84 | Toluene | 51, 65, 74, 89, **91** |
| 10 | 8.42 | 2-Ethylhexene | 55, **70**, 83, 85, 95, 112 |
| 11 | 8.50 | 1-Octene | **55**, 57, 70, 83, 97, 112, 113 |
| 12 | 10.75 | 1-Pentanol, 2-ethyl-4-methyl- | **57**, 69 83, 97, 112 |
| 13 | 11.54 | 3,7-Dimethyl-1-octene | **55**, 69, 84, 97, 111, 140 |
| 14 | 11.96 | 1-Octanol, 3,7-dimethyl- | **55**, 69, 84, 97, 112, 125, 140 |
| 15 | 12.08 | 1-Octanol, 2-butyl- | **57,** 71, 85, 97, 111, 125, 140 |
| 16 | 12.26 | 3,7-Dimethyl-1-octene | **55**, 70, 83, 97, 111, 113, 125, 140 |
| 17 | 12.45 | 4-Propylheptane / 2-Ethylhexanol | 55, **57**, 70, 83, 84, 97, 98, 111, 112 |
| 18 | 12.67 | 1-Decene | **55**, 70, 83, 97, 111, 140 |
| 19 | 12.83 | 2-Undecene, 4,5-dimethyl-, | 55, **69**, 83, 95, 98, 111, 126, 168 |
| 20 | 13.00 | 3,4-Dimethyl-1-octene | **55**, 69, 84, 97, 112, 140 |
| 21 | 13.19 | 4-Isopropylheptane | **57,** 98, 142 |
| 22 | 13.45 | 5-Tridecene, (E) | **55,** 69, 83, 97, 111, 125, 139, 154, 182 |
| 23 | 13.69 | Ethylhexanol | 55, **57,** 70, 83, 84, 98, 99, 112 |
| 24 | 15.06 | 1-Decene | **55**, 70, 83, 97, 111, 140 |
| 25 | 15.34 | Phenol | 66, **94** |
| 26 | 16.75 | Indene | 50, 63, 74, 76, 89, 109, **116** |
| 27 | 19.97 | Naphthalene, 1,4-dihydro- | 51, 64, 77, 89, 102, 115, 128, **130** |
| 28 | 20.22 | 1-Hexene, 4,4-diethyl- | **57**, 69, 99 |
| 29 | 20.57 | Naphthalene | 51, 63, 75, 102, **128** |
| 30 | 24.68 | Naphthalene, 1-methyl- | 57, 63, 71, 87, 89, 115, 138, **142** |
| 31 | 26.35 | Diphenyl | 76, 115, **154** |
| 32 | 34.33 | Anthracene | 51, 63, 76, 89, 115, 139, 152, 163, **178** |
| 33 | 35.67 | Palmitic acid, methyl ester | 60, **73**, 83, 129, 143, 171, 185, 199, 241, 284 |
| 34 | 38.41 | Oleic acid, methyl ester | **55**, 69, 83, 97, 110, 125, 141, 166, 180, 222, 264 |
| 35 | 38.77 | Stearic acid, methyl ester | 60, **73**, 83, 101, 129, 143, 157, 171, 185, 213, 227, 256, 298 |
| 36 | 43.04 | Triphenyl phosphate (TPP) | 51, 65, 77, 94, 115, 141, 169, 215, 233, 249, **326** |
| 37 | 43.97 | Phenyl palmitate | 57, 71, **94**, 109, 137, 151, 221, 239, 267, 332 |
| 38 | 46.71 | Phenyl stearate | 57, 71, **94**, 109, 136, 267, 360 |

**Table S3**: Main pyrolysis products obtained by Py-GC/MS of the investigated **Jedi plate** at their corresponding retention time (RT min) and mass to charge ratio (*m*/*z*) (base peak marked in bold).

| Peak # | RT min | Compound Name | m/z |
|---|---|---|---|
| 1 | 3.52 | Butadiene | 54 |
| 2 | 3.73 | Isoprene | 53, **67** |
| 3 | 3.90 | 1,3-Cyclopentadiene | 66 |
| 4 | 4.26 | 1-Hexene | **56**, 69, 84 |
| 5 | 5.25 | Benzene | 51, **78** |
| 6 | 7.94 | Toluene | 51, 65, 74, 89, **91** |
| 7 | 8.44 | 2-Ethylhexene | 55, **70**, 83, 112 |
| 8 | 8.69 | 3-Ethyl-3-hexene | **55,** 70, 83, 112 |
| 9 | 9.05 | 2-Heptene, 3-methyl- | 55, **70**, 83, 112 |
| 10 | 9.23 | 2-Octene, (E)- | 55, **70**, 83, 112 |
| 11 | 11.18 | Ethylbenzene | 51, 65, 77, **91**, 103, 106 |
| 12 | 12.19 | o-Xylene | 51, 65, 77, **91**, 103, 106 |
| 13 | 14.39 | 3-Chloro-3-methylheptane | 55, 63, **70**, 83, 91, 112, 119 |
| 14 | 16.02 | 1-Chloro-2-ethylhexane | **57**, 99, 121 |
| 15 | 17.21 | Ethylhexanol | 55, **57,** 70, 83, 98, 112 |
| 16 | 20.14 | Naphthalene, 1,2,3,4-tetrahydro- | 51, 65, 77, 91, **104**, 115, 128, 132 |
| 17 | 21.20 | Naphthalene | 51, 63, 75, 102, **128** |
| 18 | 23.84 | Naphthalene, 1-methyl- | 57, 63, 71, 87, 89, 115, 138, **142** |
| 19 | 24.13 | Naphthalene, 2-methyl- | 63, 115, 141, **142** |
| 20 | 24.46 | Phthalic anhydride | 50, **76**, 104, 148, 149 |
| 21 | 32.06 | 2-Ethylhexyl benzoate | 55, 70, 83, 92, **105**, 112, 123, 135, 149 |
| 22 | 35.50 | Hexadecanenitrile | **57**, 70, 83, 97, 110, 124, 138, 152, 166, 180, 194, 208 |
| 23 | 38.59 | Octadecanenitrile | **57**, 70, 97, 110, 124, 138, 152, 166, 180, 208, 222, 236 |
| 24 | 44.34 | Bis(2-ethylhexyl)phthalate (DEHP) | 57, 70, 83, 104, 121, **149**, 167, 233, 251, 279 |

**Table S4**: Main pyrolysis products obtained by Py-GC/MS of the investigated **Dia-bag** at their corresponding retention time (RT min) and mass to charge ratio (*m*/*z*) (base peak marked in bold).
